# Clinical features of freezing of gait in Parkinson's disease patients

**DOI:** 10.1002/brb3.1244

**Published:** 2019-03-09

**Authors:** Makoto Sawada, Kenji Wada‐Isoe, Ritsuko Hanajima, Kenji Nakashima

**Affiliations:** ^1^ Division of Rehabilitation National Hospital Organization Tottori Medical Center Tottori Japan; ^2^ Division of Neurology Department of Brain and Neurosciences Faculty of Medicine Tottori University Yonago Japan; ^3^ National Hospital Organization Matsue Medical Center Matue Japan

**Keywords:** fatigue, new freezing of gait questionnaire, postural instability and gait difficulty

## Abstract

**Objective:**

To clarify the clinical features of freezing of gait (FOG) in Parkinson's disease (PD) patients by classification into two groups: Clinically observed FOG (CFOG) and self‐reported FOG (SFOG).

**Methods:**

Two hundred twenty‐nine PD patients were medically examined in an examination room as well as subjected to a New Freezing of Gait Questionnaire (NFOG‐Q) and analysis of nonmotor symptoms including sleep, cognition, depression, and fatigue.

**Results:**

The prevalence of CFOG was 17.9%, while 53.7% of the patients without CFOG reported the presence of FOG via the NFOG‐Q. Univariate analysis revealed that CFOG was associated with longer disease duration, motor dysfunction, sleepiness, fatigue, and cognitive dysfunction. These symptoms, excluding akinesia, apathy, rapid eye movement (REM) sleep Behavior Disorder, and cognitive dysfunction, were also associated with SFOG. Multivariate analysis revealed that long PD duration, postural instability, and gait difficulty (PIGD), along with fatigue, were independent factors for SFOG.

**Conclusions:**

SFOG and CFOG have many common clinical features. Although the clinical relevance of SFOG remains unclear, careful attention should be paid to related features in clinical practice.

## 1 INTRODUCTION

Freezing of gait (FOG) is a major disabling motor symptom that affects the daily quality of life of Parkinson's disease (PD) patients. Prevalence of FOG increases with longer disease duration (Kalia & Lang, 2015). It has been reported that 81% of PD patients experienced FOG after a disease duration of 20 years (Hely, Reid, Adena, Halliday, & Morris, 2008). FOG in PD patients is the main cause of falling, fracture risk, and activities of daily living disability (Okuma, 2014; Okuma, Silva de Lima, Fukae, Bloem, & Snijders, 2018).

The pathogenesis of FOG is complicated and involves multiple mechanisms, including disordered limbic system and cognitive functions regulated by the basal ganglia, supplementary motor area, and cerebral cortex (Iseki et al., 2015; Okuma, 2014; Snijders et al., 2016; Teramoto, Morita, Ninomiya, Shiota, & Kamei, 2014). The association of FOG with these systems has not yet been investigated in detail.

The accurate detection of FOG is often difficult in daily clinical practice, as FOG is not often observed in outpatients, because PD patients always come to hospital in the “on” state, and some patients may not completely understand FOG well enough to ask about it. Recently, the detection of FOG by wearable devices has been attempted (Silva de Lima et al., 2017). However, this technology is still in the research stage and is not used in clinical practice. On the other hand, evaluation of FOG via questionnaire is simple and less burdensome for patients (Barthel, Mallia, Debu, Bloem, & Ferraye, 2016). High sensitivity and specificity have been reported using the New Freezing of Gait Questionnaire (NFOG‐Q), which uses a video that explains FOG (Barthel et al., 2016; Nieuwboer et al., 2009). We hypothesized that the number of PD patients with subjectively reported FOG in everyday life is more than expected and that investigation of the differences in associated factors between objectively detected FOG and subjectively reported FOG would provide some clues for elucidating the mechanisms of FOG in PD patients. This study had the following two aims. First, to identify FOG in daily practice by a clinical examination and a questionnaire. Second, to compare the clinical features (such as demographic characteristics, motor symptoms, nonmotor symptoms, cognitive function, and medication use) between FOG identified by a clinical examination and FOG identified by a questionnaire.

## 2 METHODS

### 2.1 Subjects

We recruited consecutive patients with sporadic PD in the Department of Neurology at Tottori University Hospital between July 2014 and April 2017. The diagnosis of PD was confirmed according to the United Kingdom PD Brain Bank. Regardless of the presence or absence of FOG, we investigated all of the patients who consented to our study by questionnaire and examination. Patients who were unable to walk or who had undergone functional stereotactic surgery for PD were excluded, but PD patients with dementia were investigated via responses from their families. The study was approved by the Tottori University Committee for Medical Research Ethics and followed the principles outlined in the Declaration of Helsinki.

### 2.2 Self‐reported FOG

In order to detect self‐reported FOG (SFOG), we used a self‐administered questionnaire (NFOG‐Q) (Nieuwboer et al., 2009). The range of NFOG‐Q scores was 0–28; we defined an NFOG‐Q score ≥ 1 point as SFOG‐positive.

### 2.3 Clinically observed FOG

**Figure 1:**
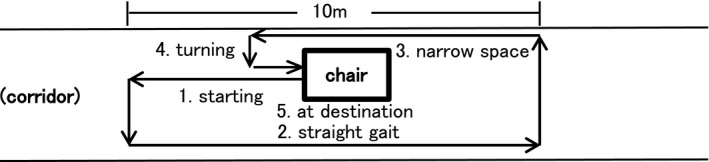
Walking route for examining clinically observed freezing of gait

After patients completed the NFOG‐Q, we performed physical examinations to assess clinically observed FOG (CFOG). We instructed patients to walk naturally through a fixed course. We created a few situations in the course that are reported to have a high probability of FOG occurrence (Nutt et al., 2011; Schaafsma et al., 2003; Snijders, Haaxma, Hagen, Munneke, & Bloem, 2012). We checked for the presence or absence of FOG at 5 points, with reference to the previous study of Schaafsma et al. (2003): (a) start hesitation, when freezing was detected as the patient initiated walking; (b) straight gait, (c) apparent hesitation in a narrow space, when FOG was noted when the patient passed through a narrow space (d) turning hesitation, when the patient's feet appeared to become stuck whilst making a turn, and (e) destination hesitation, when the patient's feet appeared to freeze as the patient approached a chair (Figure 1). We defined CFOG as “a brief, episodic absence or marked reduction of forward progression of the feet despite the intention to walk” during the patient's walk through the course (Nutt et al., 2011).

### 2.4 Assessment of motor and nonmotor symptoms

Motor symptoms were evaluated using the Unified Parkinson's Disease Rating Scale (UPDRS), Part 3. We classified Tremor, Akinesia, Rigidity, and postural instability gait difficulty (PIGD) using the results of UPDRS Part 3, in which Items 20 and 21 were for tremor, Item 22 was for rigidity, Items 23–26 and 31 were for bradykinesia, and Items 27–30 were for PIGD (Zuo et al., 2017). In addition, to assess nonmotor symptoms, we distributed another questionnaire including a Japanese version of each of the following: Geriatric Depression Scale (GDS), Apathy Scale (AS), Pittsburgh Sleep Quality Index (PSQI), (Buysse, Reynolds, Monk, Berman, & Kupfer, 1989) Japanese version of the Epworth Sleepiness Scale (JESS), (Takegami et al., 2009) REM sleep Behavior Disorder Screening Questionnaire (RBDSQ), (Stiasny‐Kolster et al., 2007) and Parkinson Fatigue Scale (PFS) (Okuma et al., 2009).

### 2.5 Information from other assessment batteries

We recorded gender, age at evaluation, age at onset of PD, disease duration, Hoehn and Yahr stage (HY), and a cognitive function battery examined at the same time, including Mini Mental State Examination (MMSE), Frontal Assessment Battery (FAB), and Montreal Cognitive Assessment (MoCA) (Nasreddine et al., 2005). In relation to MMSE and MoCA, we performed a detailed evaluation by subdomain as shown by Lessig, Nie, Xu, and Corey‐Bloom (2012). We also recorded treatment of patients with antiparkinsonian drugs, including levodopa (levodopa/carbidopa, levodopa/benserazide), entacapone, pramipexole, ropinirole, selegiline, amantadine, apomorphine, pergolide, cabergoline, bromocriptine, and rotigotine, on the date of the survey. We converted the total dose of these drugs into a levodopa equivalent daily dose (LEDD) with the formula developed by Tomlinson et al. (2010).

### 2.6 Statistical analysis

Continuous variables were checked for normality and homogeneity of variance with Shapiro–Wilk's and Levene's tests. Comparisons between groups were analyzed with Kruskal–Wallis tests with post hoc Mann–Whitney *U* tests. The chi‐square test was used to assess associations between categorical variables. Multiple regression analysis adjusted for age at evaluation and sex was performed to elucidate independent factors for the NFOG‐Q. We used the stepwise forward method, and the variables that had statistical significance in univariate analysis were chosen. Subjects were grouped into quartiles based on scores on PIGD and PFS. The quartiles for PIGD were <1 (Q1), 1–2 (Q2), 3–5 (Q3), and 6 or more (Q4). The quartiles for PFS were <31 (Q1), 32–46 (Q2), 47–56 (Q3), and 57 or more (Q4). Comparisons between groups were analyzed by Kruskal–Wallis tests with post hoc Mann–Whitney *U* tests. We used a level of 95% (*p* < 0.05) as the criterion for statistical significance. Data analysis was conducted with SPSS for Windows, version 20 (Chicago, IL).

## 3 RESULTS

### 3.1 Patients and classification of FOG

**Figure 2:**
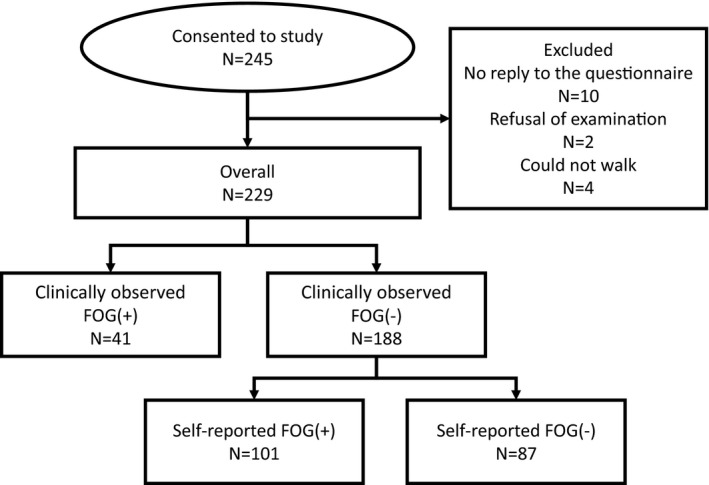
Classification of freezing of gait (FOG) in Parkinson's disease patients

In this study, we enrolled 245 patients with sporadic PD. Ten patients were excluded due to nonreply to the questionnaire, two patients were excluded due to examination refusal, and four patients could not walk. In the end, the sample comprised 229 PD patients. CFOG was observed in 41 patients. In the CFOG group, 20 patients were in the ON state, and four patients were in the OFF state at examination; 17 patients did not have wearing‐off symptoms. The most frequent freezing occurred upon turning (27 patients; 66% of objective freezers). Of the patients who did not show CFOG, 101 patients reported FOG on the NFOG‐Q, and 87 patients did not (Figure 2). In the SFOG group, 62 patients were in the ON state, and five patients were in the OFF state at examination; 23 patients did not have wearing‐off symptoms. The status of 11 patients was unknown.

### 3.2 Clinical features of CFOG and SFOG

Demographic features related to FOG are shown in Table 1. According to Kruskal–Wallis tests, we found significant differences in the NFOG‐Q, duration of disease, severity of HY stage, UPDRS Part 3 (Rigidity, Akinesia, PIGD), GDS, AS, PSQI, JESS, RBDSQ‐J, PFS, MMSE, FAB, MoCA‐J, and LEDD.

**Table 1:** Descriptive statistics

Variables		Nonfreezer	With SFOG	With CFOG	Kruskal–Wallis test
		*n* = 87	*n* = 101	*n* = 41	*p*‐value
NFOG‐Q	Median (IQR)	0	14.5 (9.0–20.0)**	20.0 (15.0–24.0)** ^,^ ##	<0.01
Demographic factors					
Male:female	*n*	31:56	44:57	18:23	N/A
Age (years)	Median (IQR)	71.0 (64.0–78.0)	71.5 (65.3–76.8)	75.0 (69.0–80.0)	0.06
PD duration	Median (IQR)	4.0 (2.0–7.0)	6.0 (2.0–10.0)**	10.0 (5.0–15.0)**	<0.01
Motor symptoms					
Hoehn‐Yahr stage	Median (IQR)	2.0 (1.0–3.0)	3.0 (2.0–3.0)**	3.0 (3.0–4.0)** ^,^ ##	<0.01
UPDRS Part 3	Median (IQR)	15.0 (8.0–22.0)	20.0 (13.25–28.0)**	26.0 (22.0–34.0)** ^,^ ##	<0.01
Tremor	Median (IQR)	1.0 (0.0–2.0)	0.0 (0.0–2.0)	1.0 (0.0–2.0)	0.72
Rigidity	Median (IQR)	4.0 (2.0–5.0)	5.0 (3.25–7.0)*	6.0 (3.0–7.0)**	<0.01
Akinesia	Median (IQR)	7.0 (4.0–10.0)	9.0 (5.0–12.75)	11.0 (8.0–15.0)** ^,^ #	<0.01
PIGD	Median (IQR)	1.0 (0.0–4.0)	3.0 (1.0–5.0)**	8.0 (5.0–9.0)** ^,^ ##	<0.01
Nonmotor symptoms					
GDS	Median (IQR)	3.5 (1.0–6.0)	4.0 (2.0–9.0)*	5.0 (3.0–8.75)	<0.05
AS	Median (IQR)	15.0 (8.0–18.0)	16.0 (12.75–19.0)	18.5 (14.0–23.0)*	<0.01
PSQI	Median (IQR)	5.0 (3.0–7.75)	7.0 (4.0–10.0)*	7.0 (5.0–10.0)*	<0.01
JESS	Median (IQR)	4.0 (2.0–7.0)	6.0 (3.0–9.75)*	6.0 (3.5–12.5)*	<0.05
RBDSQ‐J	Median (IQR)	3.0 (1.0–5.0)	3.0 (2.0–6.0)	4.0 (2.0–7.0)*	<0.05
PFS	Median (IQR)	36.0 (25.0–49.0)	49.5 (34.0–61.0)**	50.0 (42.5–64.0)**	<0.01
Cognitive function					
MMSE	Median (IQR)	29.0 (26.0–29.5)	28.0 (25.0–29.0)	26.0 (23.0–27.25)**	<0.01
FAB	Median (IQR)	15.0 (13.0–16.0)	13.5 (11.0–16.0)*	12.0 (11.0–14.0)*	<0.01
MoCA‐J	Median (IQR)	25.0 (22.0–26.0)	23.0 (10.0–26.0)	21.0 (24.75)**	<0.05
LEDD (mg)	*M* (*SD*)	271.5 (250.4)	428.9 (332.2)**	530.1 (398.6)**	<0.01

Note

Statistical analysis was performed as follows; Chi‐square test for sex. Kruskal–Wallis tests and Mann–Whitney *U* test for age, PD duration and LEDD NFOG‐Q, Hoehn–Yahr stage, UPDRS Part 3, Tremor, Rigidity, Akinesia, PIGD, GDS, AS, PSQI, JESS, RBDSQ‐J, PFS, MMSE, FAB, and MoCA‐J.

PD: Parkinson's disease; NFOG‐Q: New Freezing of Gait Questionnaire; UPDRS: Unified Parkinson's Disease Rating Scale; PIGD: postural instability and gait difficulty; LEDD: levodopa equivalent daily dose; GDS: Geriatric Depression Scale; AS: Apathy Scale; PSQI: Pittsburgh Sleep Quality Index; JESS: Japanese version of the Epworth Sleepiness Scale; RBDSQ: REM Sleep Behavior Disorder Screening Questionnaire; PFS: Parkinson Fatigue Scale; MMSE: Mini Mental State Examination; FAB: Frontal Assessment Battery; MoCA: Montreal Cognitive Assessment; SFOG: self‐reported FOG; CFOG: clinically observed FOG. N/A: not applicable.

*p < 0.05 and **p < 0.01, Mann–Whitney *U* test, PD nonfreezing versus SFOG/CFOG; ^#^ *p *< 0.05 and ^##^ *p *< 0.01, Mann–Whitney *U* test, PD SFOG versus CFOG.

Mann–Whitney tests revealed significant differences in the PD duration, Hoehn–Yahr stage, UPDRS Part 3, Rigidity, Akinesia, PIGD, AS, PSQI, JESS, RBDSQ‐J, PFS, MMSE, FAB, MoCA‐J, and LEDD between the nonfreezing group and the CFOG group. Significant differences were found in the NFOG‐Q, PD duration, HY stage, UPDRS Part 3, Rigidity, PIGD, GDS, PSQI, JESS, PFS, FAB, and LEDD between the nonfreezing group and the SFOG group. Significant differences were found with regard to the NFOG‐Q, HY stage, UPDRS Part 3, Akinesia, and PIGD between the CFOG group and the SFOG group (Table 1).

### 3.3 Independent predictors for NFOG‐Q scores

Multivariate regression analysis revealed that the PIGD, PD duration, and PFS scores were independent predictors of NFOG‐Q scores (Table 2).

**Table 2:** Predictors for SFOG

	β	*p*‐Value
PIGD	0.41	<0.01
PD duration	0.18	<0.01
PFS	0.14	<0.05

SFOG: self‐reported FOG; PD: Parkinson's disease; PIGD: postural instability and gait difficulty; PFS: Parkinson Fatigue Scale.

**Figure 3:**
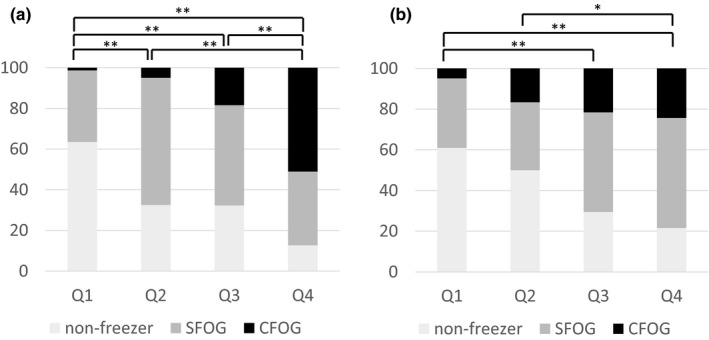
Nonfreezing and SFOG and CFOG ratio according to postural instability and gait difficulty (a) and Parkinson Fatigue Scale (b) severity. *p < 0.05, **p < 0.01. FOG: freezing of gait; SFOG: self‐reported FOG; CFOG: clinically observed FOG

We also found that the higher the PIGD, the more SFOG and CFOG increased. Post hoc tests related to PIGD revealed significance differences in group distributions between Q1 and the other grades, between Q2 and Q4, and between Q3 and Q4 (Figure 3a). Similarly, the higher the PFS, the more SFOG and CFOG were observed. Post hoc tests related to PFS revealed significance differences in group distribution between Q1 and Q3, between Q1 and Q4, and between Q2 and Q4 (Figure 3b).

## 4 DISCUSSION

We examined features and factors relating to CFOG and SFOG. Among our PD patients, 17.9% (41/229) exhibited CFOG, while 53.7% (101/188) of the remaining PD patients who did not show CFOG during the outpatient clinic were reported to have FOG according to the NFOG‐Q. Prevalence of FOG has been reported to range from 47% to 81%, depending on the detection method and the differences in target patient populations (Amboni et al., 2015; J. Hall et al., 2015; J. M. Hall et al., 2014; Macht et al., 2007; Rahman, Griffin, Quinn, & Jahanshahi, 2008; Shine et al., 2012; Snijders et al., 2012). Among these investigations, the prevalence of CFOG via examination was 48%–50% (Shine et al., 2012; Snijders et al., 2012), which was relatively smaller than estimates of SFOG (47%–81%) (Amboni et al., 2015; J. Hall et al., 2015; J. M. Hall et al., 2014; Hely et al., 2008; Macht et al., 2007; Rahman et al., 2008; Shine et al., 2012; Snijders et al., 2012). The prevalence of CFOG in this study was lower than that seen in previous reports. For our outpatients, this may be due to the fact that disease severity in our patients is relatively mild and patients often come to our hospital in the “on” state (Nieuwboer, De Weerdt, Dom, & Lesaffre, 1998). To the best of our knowledge, there is no research that has examined FOG separately (self‐reported and clinically observed) like ours. In daily clinical practice, FOG that can be detected in the examination room might be lower compared to previous reports, and a questionnaire might be more useful for the detection of FOG.

CFOG was reported to be related to long disease duration, global motor disability due to disease (severity of HY stage, UPDRS Part 3), and nonmotor dysfunctions (apathy, sleepiness, RBD, fatigue, cognitive deficits). Previous studies reported that FOG correlates with UPDRS Part 3, cognition, (Giladi et al., 2001) and RBD (Videnovic et al., 2013). Factors related to CFOG in our study are in agreement with these results. In comparison, we indicated that SFOG is related to a long disease duration, global motor disability due to disease (severity of HY stage, UPDRS Part 3), and nonmotor dysfunctions (depression, sleepiness, fatigue). From these results, there is a possibility that patients in the SFOG group may develop CFOG in the future. This is an important consideration for conducting future longitudinal studies. Accordingly, we think that it is important to properly identify the SFOG group so that interventions that may exacerbate motor or nonmotor symptoms at an inappropriate time are avoided in these patients.

We found that the PIGD, PD duration, and PFS scores were independent predictors of NFOG‐Q scores. As the PIGD and PFS became more severe, the proportion of patients with FOG increased. The PIGD disorder and longer duration of disease have been reported previously as risk factors for FOG in PD patients, (Amboni et al., 2015; Giladi et al., 2001) but there is no detailed report on fatigue and FOG as far as we know. Hagell et al. reported an association of fatigue with parkinsonism, (Hagell & Brundin, 2009) especially axial/postural/gait impairment. We previously reported an association between fatigue and gait disturbance in PD patients (Tanaka et al., 2014). There is a report showing that fatigue is related to a disturbance of the serotonergic system in PD patients (Pavese, Metta, Bose, Chaudhuri, & Brooks, 2010). Recently, involvement of nondopaminergic systems, such as the serotonergic system, in FOG of PD patients has been suggested (Devos, Defebvre, & Bordet, 2010; Martens et al., 2016; Takahashi, Tabu, Ozaki, Hamano, & Takeshima, 2019). Taken together with our results, fatigue and FOG in PD patients might be related, and common associated factors might exist. However, both fatigue and FOG occur at high rates as the disease progresses, and causal relationships with fatigue are unclear in our cross‐sectional study. Therefore, further longitudinal studies, exploring whether ratings of fatigue are important for the early detection of FOG in PD patients will be needed. Patients with PD with these factors may be cautioned as being at risk of deteriorating FOG.

One major limitation of this study is that a confirmed method for the detection of SFOG in PD patients is not fully established. In the present study, we used the NFOG‐Q, a validated questionnaire for FOG in PD with a higher accuracy for detection (Nieuwboer et al., 2009).

## 5 CONCLUSION

The frequency of objectively detected FOG is not high, while SFOG might be higher than previously considered. Our study demonstrates that PIGD, longer duration of disease, and fatigue are associated with FOG.

## CONFLICT OF INTEREST

The authors declare that they have no conflict of interest.
